# Hospitalization burden and comorbidities of patients with rheumatoid arthritis in Spain during the period 2002–2017

**DOI:** 10.1186/s12913-020-05243-0

**Published:** 2020-05-04

**Authors:** Mario Gil-Conesa, Juan Antonio Del-Moral-Luque, Ruth Gil-Prieto, Ángel Gil-de-Miguel, Ramón Mazzuccheli-Esteban, Gil Rodríguez-Caravaca

**Affiliations:** 1 Preventive Medicine Unit, Alcorcon Foundation University Hospital, Alcorcón, Madrid, Spain; 2 Preventive Medicine Department, King Juan Carlos University, Alcorcon, Madrid, Spain; 3 Rheumatology Unit, Alcorcon Foundation University Hospital, Alcorcón, Madrid, Spain

**Keywords:** Rheumatoid arthritis, CMBD, Hospital burden, Health system, Epidemiological study, Spain

## Abstract

**Background:**

Rheumatoid arthritis (RA) is a chronic autoimmune rheumatic disease that is associated with multiple comorbidities and has a significant economic impact on the Spanish health system. The objective of this study was to estimate the rates of hospitalization of rheumatoid arthritis in Spain, and describing hospitalization rates and their changing by age, region, RA variant, and when RA as a main cause of hospitalization or a comorbidity.

**Methods:**

Observational descriptive study that reviewed hospital records from the CMBD. We included all hospitalizations of patients in Spain whose main diagnosis or comorbidity in the ICD-9-CM was rheumatoid arthritis during the period of 2002–2017.

**Results:**

A total of 315,190 hospitalizations with the RA code were recorded; 67.3% were in women. The mean age of the patients was 68.5 ± 13.9 years. The median length of hospital stay was 7 days (IQR 3–11 days). In 29,809 of the admissions, RA was coded as the main diagnosis (9.4%). When RA was not coded as the main diagnosis, the most frequent main diagnoses were diseases of the circulatory system (18.9%) and diseases of the respiratory system (17.4%). The hospitalization rate during the period of 2002–2017 was 43.8 (95% CI: 43.7–44.0) per 100,000 inhabitants and constantly increased during the period. The total cost for the healthcare system was 1.476 million euros, with a median of 3542 euros per hospitalization (IQR 2646–5222 euros).

**Conclusions:**

In Spain, the hospitalization rate of patients with RA increased during the study period, despite the decrease in the hospitalization rate when RA was the main diagnosis.

## Background

Rheumatoid arthritis (RA) is a chronic inflammatory disease that preferentially affects the joints symmetrically; however, it can also damage internal organs and thus should be considered a systemic disease. Systemic involvement and comorbidities can reduce life expectancy [1].

RA is associated with multiple comorbidities and psychosocial problems, which include cardiovascular diseases, interstitial lung disease, osteoporosis, infections, fatigue and depression [2, 3] and an increased risk of early mortality [4, 5], amongst others. RA is also associated with an increased incidence of lymphomas, leukaemia and lung cancer [6]. These comorbidities are associated with a significant increase in mortality, disability and costs, which is why they are a priority addressed by the new strategies of the World Health Organization (WHO) [7].

The worldwide prevalence of RA is estimated to be 0.24%, whereas the prevalence of this disease is 0.66% in Northern Europe [8] and 0.44% in Western Europe [9]. In Spain, the prevalence of RA is 0.5% (0.8% in women and 0.2% in men), with approximately 200,000 cases and a 4:1 ratio between women and men, according to the EPISER study (National Survey on the Prevalence of Rheumatic Diseases in Spain) [10]. The incidence of RA in our country is 8.3 cases/100,000 inhabitants, which increases with age in both sexes [11]. According to the National Health Survey (ENSE 2011/2012), in the section “Chronic Problems of the Adult Population”, under the label of “Arthrosis, Arthritis and Rheumatism”, RA was classified as the primary disease in women (affecting 25.1%) and as the third most common disease in men (affecting 11.1%) [12].

In Spain, 74% of the total RA costs correspond to direct costs, and 26% correspond to indirect costs [13].

There is clearly a lack of recent studies describing the trend in rheumatoid arthritis hospitalizations in Spain, its costs and differences by age and sex, as well as its variants.

This study provides relevant information to understand the distribution of this disease in Spain, and that has been carried out with reliable and complete national data representative of the population of our country. In addition, hospitalization rates for this disease have not been presented in recent studies, making it necessary to describe the changes in its trends, as well as its cost to the health system.

We consider that it is necessary to make large sample studies in which we can consistently observe differences that can help us clarify the etiology of this complex disease.

Our study aimed to estimate the rates of hospitalization of rheumatoid arthritis in Spain during the period 2002–2017 and describing hospitalization rates by year, age, region, RA variant, and when RA as a main cause of hospitalization or a comorbidity. As secondary objectives, we also described main diagnosis when RA was not the cause of hospitalization, and its principal comorbidities when it was the main cause, describing as well the costs of RA as a global and individually.

## Methods

### Study design

An observational descriptive study was performed using data from the Minimum Basic Data Set (CMBD) of the Ministry of Health, Social Services and Equality of Spain. The CMBD is a compulsory registry for both public and private hospitals that provides statistical knowledge of hospital morbidity. This information helps in the planning and evaluation of health systems [14, 15] and includes an estimated 98% of hospital admissions, covering 99.5% of the Spanish population [16]. We worked with episodes of hospitalization. All patients were identified by their main diagnosis code and their medical record number, excluding second episodes for individual analysis but including them in the total number of episodes studied.

The main inclusion criterion in the study was the diagnosis of RA at hospital discharge (code 714.0 or its variants) and all the patients with RA admitted to the hospital were captured by the coding system.

### Sampling and variables

Our registry included 315,190 hospital admissions from a period of 16 years (from January 1, 2002 to December 31, 2017). A total of 69 variables were collected; from these, this study analysed sex, age, year, average stay, cost of hospitalization, autonomous community of admission, primary diagnosis and secondary diagnoses (up to 13 diagnoses). The main diagnostic variable was considered the reason for hospitalization, and the rest of the diagnostic variables were assessed as comorbidities. The cost of each episode of RA and its variants was calculated by the Ministry of Health and it is included in the CMBD database and it corresponds to the estimated average cost for each Group Related to the Diagnosis (DRG) updated for the year of reference. The calculated cost took into account each severity level of the episode.

RA and its variants were coded in one or more of 14 diagnostic variables based on the Ninth Edition of the International Classification of Diseases (ICD-9-CM). According to this classification, RA and all its variants were registered with the code 714 followed by one or two figures.

The patients were grouped into the following age ranges: under 20 years old, 20–29 years old, 30–39 years old, 40–49 years old, 50–59 years old, 60–69 years old, 70–79 years old, 80–89 years old and over 89 years old. To calculate the hospitalization rates (by year, age and autonomous community), the number of hospitalizations of RA patients was used as the numerator and the official population figure of the National Institute of Statistics [17] was used as the denominator, calculated per 100,000 inhabitants.

### Statistical analysis

Qualitative variables were described with their frequency distribution and were compared using Pearson’s Chi-square test or Fisher’s exact test if their application criteria were not met. Quantitative variables were described with the mean and standard deviation (SD) or the median and interquartile range (IQR) if they did not follow normal distributions and were compared using Student’s t-test when the data followed a normal distribution or with the Mann-Whitney U test otherwise. Quantitative variables with more than two categories were compared using analysis of variance (ANOVA), and when the application conditions were not met, the non-parametric Kruskal-Wallis test was used. The statistical analysis was performed using the statistical program SPSS 22.0 (SPSS Inc., Chicago, IL). Statistically significant differences were those with *p* < 0.05; all estimates were described with their 95% confidence intervals. Patient information was anonymized before performing the analysis.

## Results

A total of 315,190 hospitalizations were recorded for RA patients, both in primary and secondary diagnosis, during the period of 2002–2017. Of these admissions, 211,967 were women (67.3%), and 103,199 were men (32.7%). The mean age of the patients was 68.5 ± 13.9 years, corresponding to 68.2 ± 14.5 years in women and 69.1 ± 12.6 years in men (*p* > 0.05). The median length of hospital stay over the entire study period was 7 days (IQR 3–11 days); the length of stay was significantly higher in 2002 (8 days, IQR 4–13 days) compared to 2017 (6 days, IQR 3–10 days) (*p* < 0.05). In 2002, the cost of hospitalization for RA patients was 38 million euros, with a median hospitalization cost per admission of 2314 euros (IQR 2032–3417 euros), whereas the total cost was 142 million euros in 2017, with a median hospitalization cost per admission of 4396 euros (IQR 3384–5945 euros) (*p* < 0.05). The overall cost of hospitalizations for patients with RA in the Spanish Health System was 1.476 million euros during the period of 2002–2017, with a median hospitalization cost per admission of 3542 euros (IQR 2646–5222 euros).

### Hospitalization rates by sex, age, year and diagnosis

**Fig. 1:**
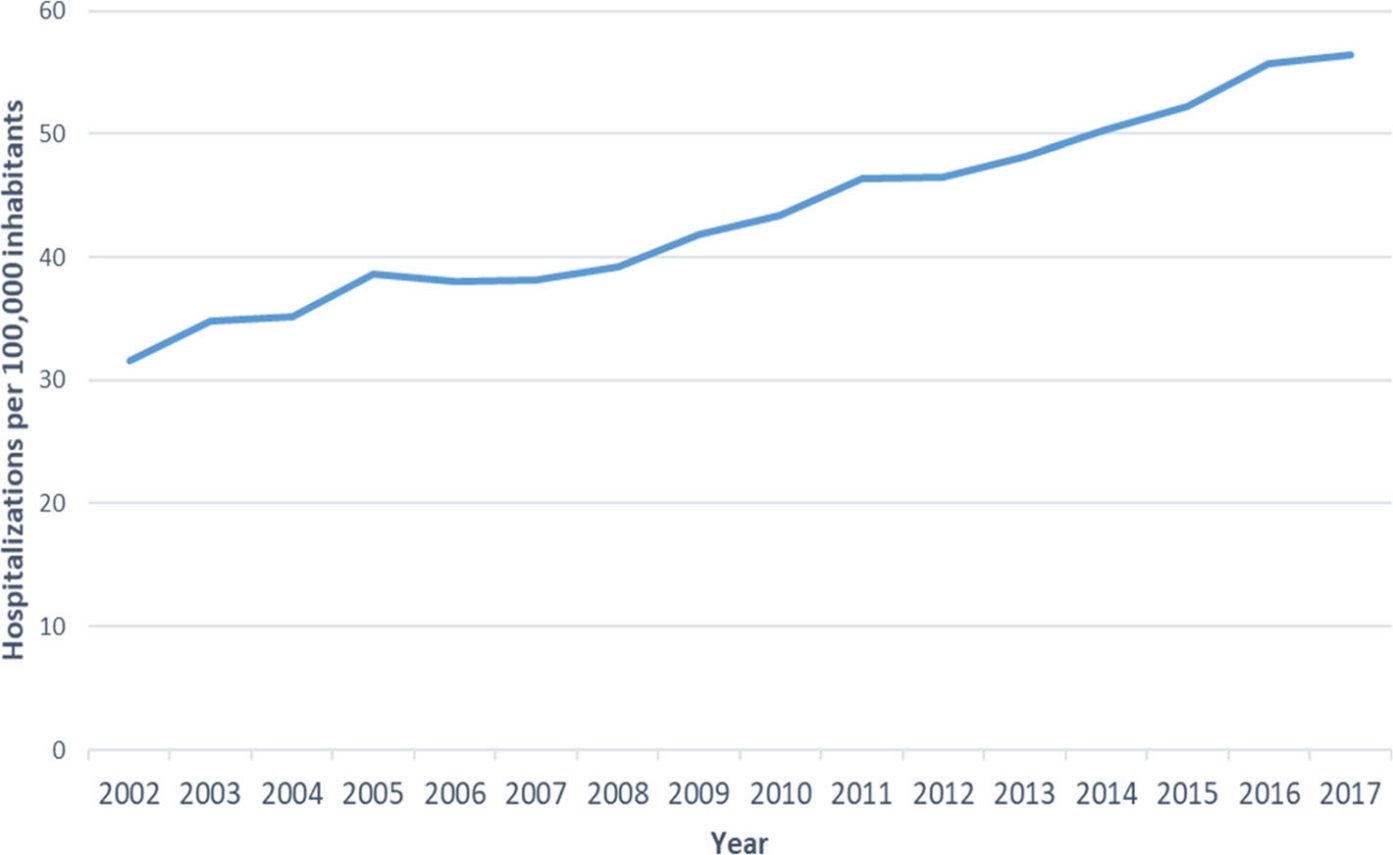
Annual hospitalization rates for rheumatoid arthritis in Spain (2002–2017)

The hospitalization rate in Spain for RA during the period of 2002–2017 was 43.8 (95% CI: 43.7–44.0) hospitalizations per 100,000 population-years. By sex, the rate of hospitalization was 58.1 (95% CI: 58.0–58.3) per 100,000 population-years in women and 29.1 (95% CI: 29.0–29.3) in men (*p* < 0.05). When stratified by age, the lowest hospitalization rate corresponded to those under 20 years old: 0.53 (95% CI: 0.49–0.56) per 100,000 population-years, and the highest corresponded to those between 80 and 90 years old: 219.0 per 100,000 population-years (95% CI: 217.5–220.6) (Table 1). When comparing the annual rate of hospitalization without differentiating whether RA was coded as the main diagnosis or a comorbidity, there was an annual increase in this rate from 31.6 per 100,000 inhabitants in 2002 to 56.3 per 100,000 inhabitants in 2017 (*p* < 0.05) (Fig. 1).

**Table 1:** Hospitalization rates for rheumatoid arthritis in Spain (2002–2017) by age. Hospitalization rate per 100,000 inhabitants

Age	Rate	95% CI
< 20 years	0.53	0.49–0.56
20–29 years	2.93	2.83–3.04
30– 39years	7.93	7.78–8.08
40–49 years	17.98	17.76–18.21
50–59 years	45.95	45.62–46.28
60–69 years	101.24	100.50–102.04
70–79 years	183.29	182.31–184.36
80–89 years	219.08	217.59–220.62
> 89 years	136.58	133.73–139.65
Total	43.88	43.77–44.00

The overall hospitalization rates during the study period were 4.1 (95% CI: 4.0–4.2) per 100,000 inhabitants when RA was coded as the main diagnosis and 39.7 (95% CI: 39.6–39.8) when it was a comorbidity at admission. The highest annual hospitalization rate for RA as the main diagnosis was 8.1 (95% CI: 7.9–8.2) per 100,000 inhabitants in 2003, whereas the lowest was 1.9 (95% CI: 1.8–2.0) in 2017; overall, there was a declining trend since 2003. Conversely, the data displayed an increasing trend of annual hospitalization rates when RA was a comorbidity; the lowest rate was in 2002: 24.5 (95% CI: 24.1–24.9) per 100,000 inhabitants, whereas the highest was in 2017: 54.5 (95% CI: 54.0–54.9) per 100,000 inhabitants (Table 2).

**Table 2:** Annual hospitalization rates in Spain (2002–2017) for rheumatoid arthritis as the main diagnosis or comorbidity. Annual hospitalization rates per 100,000 inhabitants^a^

Year	RA as main diagnosis		RA as comorbidity	
	Rate	95% CI	Rate	95% CI
2002	7.11	6.97–7.25	24.50	24.08–24.92
2003	8.10	7.98–8.22	26.67	26.25–27.10
2004	7.42	7.28–7.55	27.79	27.36–28.22
2005	7.10	6.97–7.24	31.57	31.13–32.02
2006	5.70	5.55–5.85	32.37	31.93–32.82
2007	4.28	4.14–4.43	33.90	33.46–34.34
2008	4.23	4.08–4.37	35.00	34.56–35.44
2009	3.82	3.69–3.96	37.99	37.54–38.44
2010	3.38	3.25–3.52	39.99	39.55–40.44
2011	3.34	3.20–3.47	43.08	42.63–43.53
2012	2.78	2.64–2.90	43.71	43.26–44.15
2013	2.56	2.44–2.68	45.66	45.21–46.11
2014	2.18	2.06–2.30	48.21	47.76–48.66
2015	2.09	1.98–2.21	50.12	49.67–50.57
2016	2.03	1.91–2.15	53.74	53.29–54.19
2017	1.93	1.82–2.05	54.45	54.00–54.91
Total	4.15	4.11–4.19	39.73	39.62–39.84

^a^*RA* rheumatoid arthritis

### Rheumatoid arthritis as the main diagnosis or comorbidity on admission

When RA was the main diagnosis, a total of 29,809 hospitalizations were recorded during the study period, with a hospitalization rate of 4.1 (95% CI: 3.9–4.3) per 100,000 population-years. The mean age of the patients admitted was 60.4 ± 14.7 years, and 71.6% of the patients were women. The average hospital stay was 7.1 ± 8.3 days, and the average cost per hospitalization was 3503 ± 2224 euros. RA (code 714.0) was classified as the main diagnosis in 9.2% of cases, followed by rheumatic lung and non-specific inflammatory polyarthropathy in 0.1 and 0.03% of cases, respectively. The rest of the variants of RA were classified as the main diagnosis in less than 0.03% of cases (Table 3). Amongst the 285,381 hospitalizations of patients with RA as a comorbidity, the most frequent causes of admission were diseases of the circulatory system (*n* = 54,123, 18.9%), diseases of the respiratory system (*n* = 49.876, 17.4%) and diseases of the osteomyoarticular system and connective tissue (*n* = 32.003, 11.2%) (Table 4).

**Table 3:** ICD-9-CM codes of rheumatoid arthritis and its variants when rheumatoid arthritis was coded as the main diagnosis^a^

Type of rheumatoid arthritis	Code	Principal diagnostic	
		N	%
Rheumatoid arthritis	714.0	29,059	9.22%
Felty syndrome	714.1	37	0.01%
RA with visceral or systemic implication	714.2	40	0.01%
RA youth polyarticular chronicle	714.30	78	0.02%
Acute youth polyarticular RA	714.31	19	0.01%
Oligoarticular or pauciarticular juvenile RA	714.32	11	0.003%
Monoarticular youth RA	714.33	12	0.004%
Postrheumatic arthropathy chronicle	714.4	19	0.01%
Rheumatic lung	714.81	412	0.13%
Other inflammatory polyartopathies	714.89	39	0.01%
Inflammatory non-specific polyartropathy	714.9	83	0.03%
Total RA		29,809	9.46%
Other pathologies		285,381	90.54%
Total		315,190	100%

^a^*RA* rheumatoid arthritis

**Table 4:** Causes of hospitalization when rheumatoid arthritis was not coded as the main diagnosis

ICD-9-MC codes	N	%
1. Infectious and parasitic diseases (001–139)	7447	2.61%
2. Neoplasms (140–239)	20,514	7.19%
3. Endocrine, metabolic, and nutritional diseases and immune disorders (240–279)	5500	1.93%
4. Diseases of the blood and haematopoietic organs (280–289)	5434	1.90%
5. Mental, behavioural and neurodevelopmental disorders (290–319)	2429	0.85%
6. Diseases of the nervous system and the sense organs (320–389)	6726	2.36%
7. Diseases of the circulatory system (390–459)	54,123	18.97%
8. Diseases of the respiratory system (460–519)	49,876	17.48%
9. Diseases of the digestive system (520–579)	31,864	11.17%
10. Diseases of the genitourinary system (580–629)	15,675	5.49%
11. Complications of pregnancy, childbirth and puerperium (630–679)	3466	1.21%
12. Diseases of the skin and subcutaneous tissue (680–709)	4915	1.72%
13. Diseases of the osteomyoarticular system and connective tissue (710–739)	32,003	11.21%
14. Congenital anomalies (740–759)	383	0.13%
15. Signs, symptoms and ill-defined states (780–799)	12,385	4.34%
16. Injuries and poisoning (800–999)	27,579	9.66%
17. Factors that influence the state of health and contact with health services (V01-V89)	4922	1.72%
18. Lost to follow-up (ZZZ.ZP)	140	0.05%
Total	285,381	100%

### Regional rates of hospitalization for rheumatoid arthritis in Spain

**Fig. 2:**
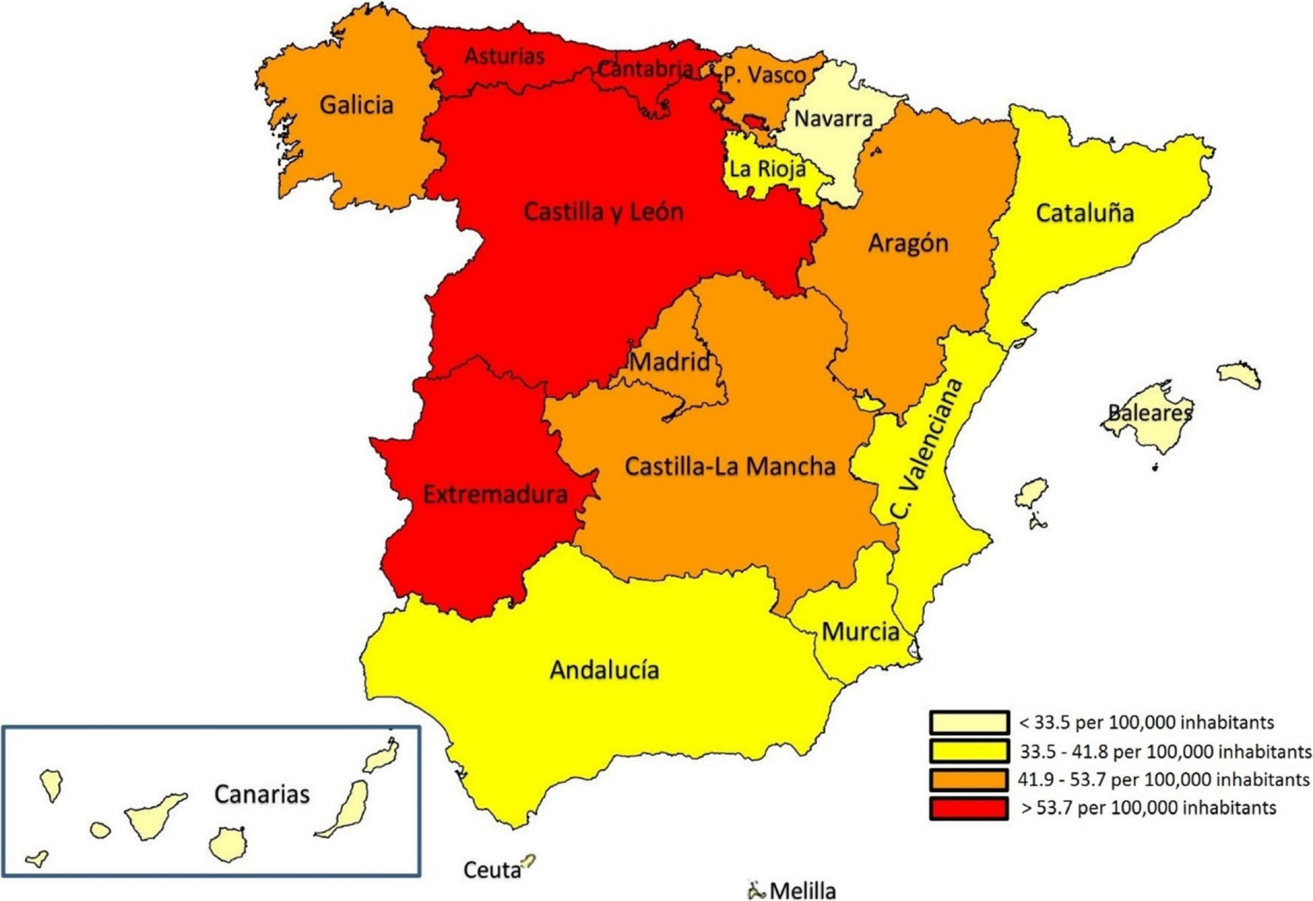
Hospitalization rates for patients with rheumatoid arthritis, by autonomous community, during the period of 2002–2017. Freely available, depicted by Flourish® public access

In Spain, the highest rates of hospitalization for RA per 100,000 population-years by autonomous communities (as the main diagnosis or comorbidity) were found in Castilla y León (69.9), Cantabria (69.9), Asturias (62.1) and Extremadura (55.8). By contrast, the lowest rates were found in Melilla (16.7), Ceuta (17.1), Canary Islands (27.9) and Balearic Islands (30.1) (Fig. 2).

## Discussion

The study carried out by the EPISER research group [18] established a population sample studied through surveys, however, our study has objective data from hospitals throughout the country, so we consider it a necessary update of the costs that this disease entails, as well as the hospitalization data of rheumatoid arthritis in the 16 years studied.

We have found an increase in the hospitalization rate for this disease during the period studied, and that seems to be in accordance with what has been done by other authors in similar conditions [19].

In our study, the rate of hospitalization for RA in women seems to be much higher than that for men what seems to be in the same direction as observed by other studies [18] [20]; furthermore, the rate of hospitalization increased with age, reflecting a trend that is consistent with the literature [10, 11].

The hospital stay has been significantly reduced during the period studied from 8 to 6 days, despite which, the total cost of rheumatoid arthritis has increased. This is due to the increase in hospitalizations for this disease and the new biological medications, which have significantly increased the treatment of this disease [20].

The finding in our study of a decrease in the annual hospitalization rate when RA is coded as the main diagnosis (and therefore considered the cause of hospitalization) could be as well result of the recent widespread use of new therapies with drug modifiers such as disease and biological agents [21]. The increase in the hospitalization rate when RA was coded as a comorbidity could be explained by the increase in the pluripathology and chronicity in patients affected by this disease for years [22].

The main causes of hospitalization in patients with RA were diseases of the circulatory, respiratory, osteomyoarticular and connective tissue systems, which constituted almost 50% of all admission pathologies. This corroborates what has been published in several studies demonstrating that cardiovascular diseases are one of the main comorbidities that accompany RA [8, 21–23]. In our study, only 2.6% of RA patient admissions were due to infections; however, in the literature, the recurrence of infections that accompany patients with this disease has been noted [23], particularly opportunistic infections such as herpes zoster or tuberculosis [24, 25].

In our study, the association of episodes with gastrointestinal diseases was also frequent, which has been shown to be consistent with other data in the literature and seems to have been increased in relation to the evolution of RA treatment [20, 23].

Patients with RA also report a significant worsening of their quality of life when they have two or more associated comorbidities [26].

In Spain, the highest hospitalization rates for RA were registered in the autonomous communities of the centre and north-west of the peninsula. The lowest rates were found in Melilla, Ceuta and both archipelagos (Canary and Balearic Islands) and in the regions located in the Mediterranean climatic zone (with the exception of Navarra).

The literature also indicates that there is a difference in the incidence of RA and its exacerbations between populations within the same country, likely because of variations in climate, environmental exposure, genetic factors and behavioural factors, amongst others [27, 28].

The limitation of this study derives from the use of a secondary information source. To improve the analysis of big data will be a long-term commitment for institutions and health authorities, not without risk or complexity, because they do not yet know all the possibilities that technologies and techniques can offer around the management and large-scale data analysis [29]. The quality of the CMBD depends on the clinical history and its codification, which leads to variability between hospital centres. However, since 2001, quality control measures have been developed to assess the internal validity of the database and improve it [30]. We have added information from other previous studies on disease burden that we have taken into account in our conclusions [31] A strength of our work is filling knowledge gaps in the field of rheumatological diseases especially hospitalization rates and their differences by age, sex and region, as well as their evolution and cost to our healthcare system. Therefore, we consider this study to be novel and of great interest.

## Conclusion

The epidemiology reported in our work follows the line of other international studies on RA and its variants. The rate of hospitalization of patients with RA increased during the period of 2002–2017 despite the decrease in the rate of RA as the main diagnosis and the use of biological therapies and more aggressive strategies to control the disease. The observed increase in hospitalization and the cost of this disease, shows that studies of incidence of the disease of larger size should be carried out to help establish the risk factors and associated health costs.

Therefore, this work is open to future lines of research through the exploitation of the CMBD and other databases that allow us to collect more information on RA to evaluate, manage and plan the processes related to this pathology in our environment.

## Data Availability

Public access to the database is closed. The data sets used and/or analysed during the current study are available from the corresponding author on reasonable request.

## Abbreviations

CMBD: Minimum Basic Data Set
CI: Confidence intervals
RA: Rheumatoid arthritis
